# Water Uptake Behavior and Young Modulus Prediction of Composites Based on Treated Sisal Fibers and Poly(Lactic Acid)

**DOI:** 10.3390/ma9050400

**Published:** 2016-05-21

**Authors:** Ander Orue, Arantxa Eceiza, Cristina Peña-Rodriguez, Aitor Arbelaiz

**Affiliations:** Materials + Technologies Group, Engineering College of Gipuzkoa, University of the Basque Country UPV/EHU, Donostia-San Sebastian 20018, Spain; ander.orue@ehu.eus (A.O.); arantxa.eceiza@ehu.eus (A.E.); cristina.pr@ehu.eus (C.P.-R.)

**Keywords:** lignocellulosic fiber, composites, surface modification, water uptake

## Abstract

The main aim of this work was to study the effect of sisal fiber surface treatments on water uptake behavior of composites based on untreated and treated fibers. For this purpose, sisal fibers were treated with different chemical treatments. All surface treatments delayed the water absorption of fibers only for a short time of period. No significant differences were observed in water uptake profiles of composites based on fibers with different surface treatments. After water uptake period, tensile strength and Young modulus values of sisal fiber/poly(lactic acid) (PLA) composites were decreased. On the other hand, composites based on NaOH + silane treated fibers showed the lowest diffusion coefficient values, suggesting that this treatment seemed to be the most effective treatment to reduce water diffusion rate into the composites. Finally, Young modulus values of composites, before water uptake period, were predicted using different micromechanical models and were compared with experimental data.

## 1. Introduction

One of the main disadvantages of lignocellulosic fibers is their hydrophilic nature, which resulted in low compatibility with hydrophobic polymeric matrices during composite fabrication. Due to fiber hydrophilic character, swelling by water uptake could lead to microcracking of the composite and degradation of mechanical properties [1]. Therefore, water uptake is one of the most serious problems that prevents a wider use of lignocellulosic fiber in composites [1,2]. In a previous work [3], it was observed that the surface energy of poly(lactic acid) (PLA) was close to sisal fiber, resulting in good wettability with poly(lactic acid) matrix. Raj *et al.* [4] suggested that the adhesion between fiber and PLA matrix was promoted by physical interactions, such as van der Waals forces and hydrogen bonds. However, the stress transfer from PLA matrix to lignocellulosic fiber was poor [5] and several fiber surface treatments were used to improve fiber/PLA matrix interfacial adhesion [3,6,7,8,9,10,11,12,13]. For example, Rajesh *et al.* [14] treated sisal fibers with 10 wt % NaOH solution followed by H_2_O_2_. After that, they prepared PLA/sisal fiber composites by an injection molding process. They observed that composites based on treated sisal fibers showed higher strength and modulus values than composites based on untreated fibers. They suggested that fiber/matrix interfacial adhesion was improved after fiber surface treatment. Rajesh *et al.* [14] also carried out water absorption measurements of composites, and they observed that the water absorption for all composites increased as a function of sisal fiber due to the hydrophilic nature of natural fibers. Furthermore, they observed that the water absorption percentage decreased in composites with successive alkali treated sisal fiber. They reported that after alkali treatment sisal fibers became more hydrophobic. Jiang *et al.* [15] treated sisal fibers with 10 wt % NaOH solution, and they prepared treated sisal fiber/PLA composites by compression molding. They observed that composites based on treated sisal fibers did not offer good mechanical properties as a result of a poor fiber/matrix interfacial adhesion and an inadequate dispersion of fibers in PLA matrix.

The main highlights of this work compared to the previous paper are the water uptake behavior of the PLA/sisal composites and its effect on composites tensile properties as well as the use of different models to predict the tensile modulus. In the present study, sisal fibers were treated with alkali, silane and combination of both treatments. The effect of surface treatments on water uptake behavior of composites based on PLA matrix and untreated and treated fibers were carried out. The diffusion coefficients of composites were determined and the effects of water uptake on the mechanical properties of composites were studied. Moreover, the morphology of fractured surface of composites after water uptake period was observed by scanning electron microscopy (SEM). On the other hand, different micromechanical models were used to predict the Young modulus values of sisal fiber/PLA composites before the water uptake period, and obtained theoretical values were compared with the experimental data.

## 2. Experimental

### 2.1. Materials

PLA (IngeoTM, 2003D) provided by NatureWorks LLC (Minnetonka, MN, USA) was used as a matrix. According to the supplier, the D-isomer content of the PLA was 4%, with a melt flow index of 6 g/10 min at 210 °C and a density of 1.24 g/cm^3^. The melting temperature range of this PLA is 145–160 °C. Sisal fibers used in this work were kindly supplied by Celulosa de Levante S.A. (Tortosa, Spain). The chemical composition of untreated and treated sisal fibers was studied in a previous work [3]. Fibers were chopped to a length of approximately 40 mm and the diameter values varied from 100 to 200 μm. Sodium hydroxide pellets, supplied by Panreac (Castellar del Vallés, Spain), and 3-(2-aminoethyl amino) propyltrimethoxy silane, supplied by Sigma Aldrich (San Luis, AZ, USA) were used as fiber surface modifiers. Other reagents employed were glacial acetic acid supplied by Panreac.

### 2.2. Fiber Surface Treatments

All fiber surface treatments were carried out following the conditions described in previous works [3,5]. For alkali treatment, sisal fibers were pre-treated with 2 wt % NaOH solution and after this, fibers were treated with 7.5 wt % NaOH solution under reflux. Silane treatment was carried out soaking sisal fibers in 3-(2-aminoethyl amino) propyltrimethoxy silane aqueous solution (2% *v*/*v*) under continuous stirring for 3 h. Then, wet fibers were kept in air for three days before drying at 100 °C. Finally, when a combination of both treatments was applied to sisal fibers, alkali treated fibers were further modified by the silane chemical agents. The same conditions described previously for alkali and silane treatment were used.

### 2.3. Compounding and Processing of Composites

**Figure 1:**
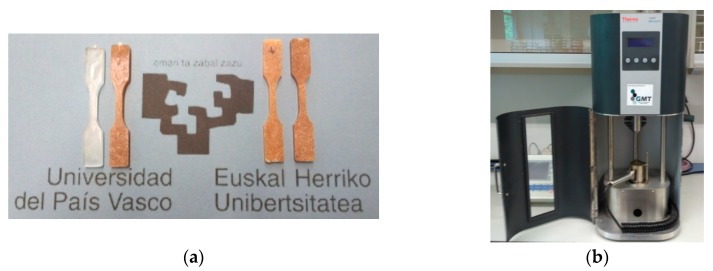
(**a**) Image of tensile specimens obtained after injection process; and (**b**) set-up machine used.

Compounding and processing conditions of composites were the same as used in the previous work [5]. Briefly, compounding was carried out in a melt mixer HAAKE Rheomix 600 (Thermo Scientific, Karlsruhe, Germany) with two Banbury rotors, and a mixing temperature of 185 °C was selected. The loading of sisal fibers varied from 20 wt % to 40 wt %. After the mixture was pelletized, the molding of pellets was carried out in a HAAKE Minijet II (Thermo Scientific, Karlsruhe, Germany) injection machine and dog bone specimens (ASTM-D-638-10 type V) were molded. Figure 1 shows tensile specimens obtained after injection process and the set-up machine used in the work.

### 2.4. Contact Angle Measurements

Contact angle values of neat PLA matrix and untreated and treated sisal fibers were measured with OCA 20 (Data Physics Instruments, Filderstadt, Germany) using HPLC water as test liquid. A controlled amount of sisal fiber was compressed in a mold to obtain disc geometry and a water droplet was deposited on the surface. Images were recorded as soon as the droplet was deposited on the surface and also every two seconds until the droplet was totally absorbed.

### 2.5. Water Uptake

In order to study the water uptake process, 63.5 mm length dog bone specimens with a narrow section of 3.18 × 3.29 mm^2^ were immersed in distilled water at 28 °C. Weight gain due to absorbed water was periodically measured taking samples out from water. Before weighing, the wet surface of the sample was quickly dried, weighed and then samples were again immersed in water. This process continued during 209 days and the water uptake at time *t* was calculated using Equation (1) [1]:

(1) $$\Delta m\;\left(t\right)=\left(\frac{w_{t}-w_{0}}{w_{0}}\right)\times 100$$

where *w*_0_ is the initial weight of specimen and *w_t_* is the weight of wet specimen at time t.

Diffusivity was analyzed with the hypothesis of a Fickian mechanism. A one-dimensional approach was followed for the determination of the diffusion coefficient, *D*, which can be calculated from Equation (2) [1,16]:

(2) $$D=\pi \;{\left(\frac{d\theta }{4\Delta m\left(\infty \right)}\right)}^{2}$$

where θ is the slope of the linear portion of the sorption curves, Δm(∞) is equilibrium water uptake value and *d* the initial sample thickness. The specimens used to determine the diffusion coefficient were normally of finite dimensions, and a correction for the effect of diffusion through the edges can be made according to Equation (3) for rectangular specimens:

(3) $$D_{c}=D{\left(1+\frac{d}{h}+\frac{d}{w}\right)}^{-2}$$

where *D*_c_ is the corrected diffusion coefficient and *h* and *w* are sample length and width, respectively. This equation is based on the assumption that the diffusion rates are the same in all directions [17]. Although tensile samples are not rectangular because they have grips that lead to water absorption, for simplicity, tensile specimens were considered as rectangular with 9.53 and 63.5 mm width and length, respectively.

#### 2.5.1. Mechanical Properties of Composites

Tensile tests of PLA and composites were performed using Insight 10 (MTS Company, Eden Prairie, MI, USA) with a load cell of 10 kN with accuracy of 0.5% and at 5 mm/min deformation rate. Tensile strength, strain at break, and Young modulus (using the video extensometer) values of composites before and after the water uptake period were determined.

#### 2.5.2. Scanning Electron Microscopy

SEM was used to analyze the fracture surfaces of neat PLA polymer and composites based on untreated and treated sisal fibers after water uptake period. SEM micrographs were performed by JSM-6400 (JEOL, Tokyo, Japan) equipment with a wolframiun filament operating at an accelerated voltage of 10 kV. All samples were coated with chromium using a Quorum Q150 TES metallizer (Ashford, UK).

### 2.6. Analysis of Young Modulus

The Young modulus of short fiber reinforced thermoplastics can be experimentally determined or estimated using different micromechanical models. Micromechanical models are based among other issues on the individual components properties of composite, such as elastic modulus of fiber (*E*_f_) and matrix (*E*_m_); the relative volume fraction of fiber $\left({Ø}_{f}\right)$ and matrix $\left({Ø}_{m}\right)$; length (*l*), diameter (*d*) and aspect ratio (*l*/*d*) values of fibers [18]. Volume fractions were determined from the weight fractions and the densities of each component measured in the previous work [5]. There are a wide variety of theoretical models to predict the elastic properties of filler reinforced composites. In this study, Hill [19] (Equation (4)), Halpin–Tsai [20] (Equation (7)) and shear-lag [21] (Equation (12)) equations were used.

#### 2.6.1. Hill Equation

(4) $$E_{c}=\frac{E_{L}+E_{T}}{2}$$

where *E*_L_ and *E*_T_ are longitudinal and transverse moduli:

(5) $$E_{L}=E_{f}\emptyset_{f}+E_{m}\emptyset_{m}$$

(6) $$E_{T}=\frac{E_{f}E_{m}}{E_{m}\emptyset_{f}+E_{f}\emptyset_{m}}$$

#### 2.6.2. Halpin–Tsai Equation

(7) $$E_{c}=\frac{3}{8}E_{L}+\frac{5}{8}E_{T}$$

In this case, longitudinal (*E*_L_) and transverse (*E*_T_) moduli are given by Equations (8) and (9):

(8) $$E_{L}=E_{m}\left[\frac{1+2\left(l/d\right)\eta_{L}\emptyset_{f}}{1-\eta_{L}\emptyset_{f}}\right]$$

(9) $$E_{T}=E_{m}\left(\frac{1+2\eta_{T}\emptyset_{f}}{1-\eta_{T}\emptyset_{f}}\right)$$

The constants η_L_ and η_T_ take the following expression (Equations (10) and (11)):

(10) $$\eta_{L}=\frac{\left(E_{f}/E_{m}\right)-1}{\left(E_{f}/E_{m}\right)+\left(2l/d\right)}$$

(11) $$\eta_{T}=\frac{\left(E_{f}/E_{m}\right)-1}{\left(E_{f}/E_{m}\right)+2}$$

where *l* and *d* refer to the length and diameter of the fiber after processing, respectively.

#### 2.6.3. Shear-Lag Analysis

(12) $$E_{c}=E_{f}\left(1-\frac{\tan h\left(\frac{\eta l}{2}\right)}{\frac{\eta l}{2}}\right)\emptyset_{f}+E_{m}\left(1-\emptyset_{f}\right)$$

In Equation (12), the shear-lag parameter (η) derived by Nairn [22] is given by the following equation (Equation (13)):

(13) $$\eta ={\left\{\frac{2}{{r_{f}}^{2}E_{f}E_{m}}\left\{\frac{E_{f}\emptyset_{f}+E_{m}\left(1-\emptyset_{f}\right)}{\frac{\left(1-\emptyset_{f}\right)}{4G_{f}}+\frac{1}{2G_{m}}\left[\frac{1}{\left(1-\emptyset_{f}\right)}\ln(\frac{1}{\emptyset_{f}}\right)-1-\frac{\left(1-\emptyset_{f}\right)}{2}]}\right\}\right\}}^{1/2}$$

where *l* and *r*_f_ are the length and radius of sisal fibers after processing, and *G*_f_ and *G*_m_ refers to shear modulus of the fiber and matrix, respectively. The values used for *G*_f_ and *G*_m_ were 1.10 and 1.29 GPa, respectively [23,24].

## 3. Results and Discussion

### 3.1. Contact Angle and Water Drop Absorption

**Figure 2:**
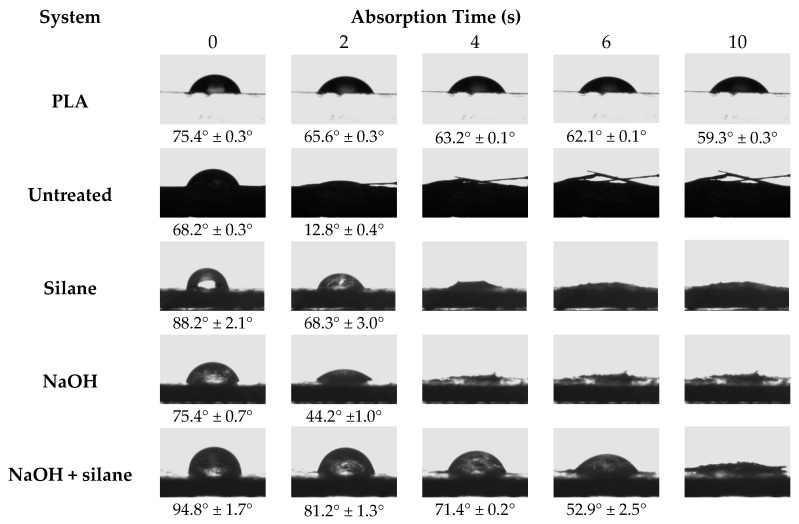
Contact angle values and the absorption of a water drop in neat poly(lactic acid) sample surface and in compressed disc geometry surfaces of sisal fibers at different intervals of time.

Figure 2 shows the contact angle values and the absorption of a water drop in neat PLA sample surface and in compressed disc geometry surfaces of sisal fibers. As reported in a previous work, the silane treatment increased the hydrophobic character of sisal fibers resulting in the highest contact angle value [3]. However, obtained images showed that all fiber surface treatments delayed the water absorption only for a short time period. After 10 s, the water drop was completely absorbed by untreated and treated sisal fibers. Although the fiber surface was modified after chemical treatments, possibly the water could penetrate through the internal porous structure, called the lumen, being probably the main reason for absorbing water [25].

### 3.2. Water Uptake of Composites

**Figure 3:**
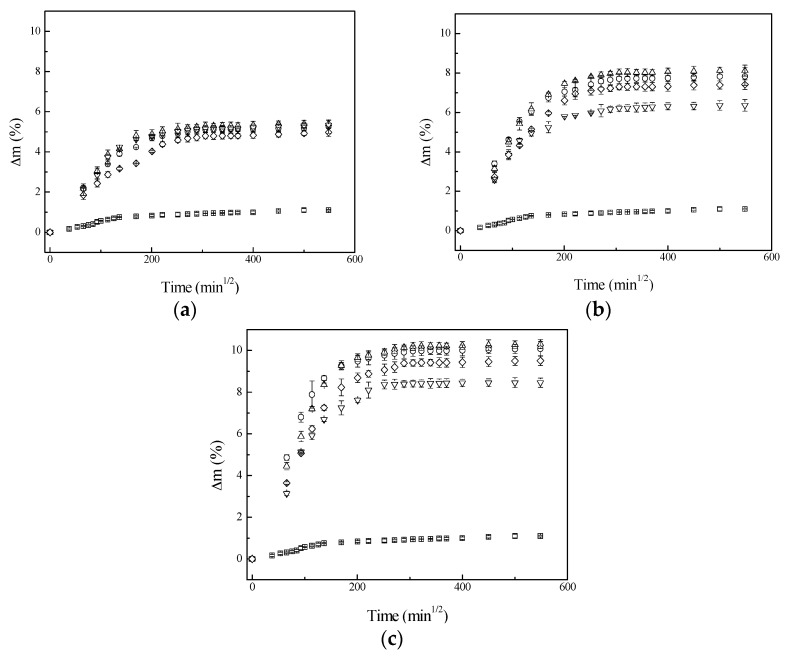
Water uptake profiles for composites as a function of square root of immersion time for different fiber surface treatments and fiber content: (**a**) 20 wt %; (**b**) 30 wt %; and (**c**) 40 wt %. (▫) Poly(lactic acid), (◦) Untreated, (Δ) Silane, (∇) NaOH and (◊) NaOH + silane.

One serious handicap related to the use of lignocellulosic fibers in composite materials is their sensitivity to water, which can reduce dramatically composite mechanical performances [1,11]. The evolution of the water absorption profiles as a function of square root of immersion time for different chemical treatments and fiber contents is showed in Figure 3a–c. In all samples, an initial linear relationship between water uptake and square root of time is observed, followed by plateau saturation, indicating a typical Fickian behavior. Due to moderate polarity induced by the presence of ester bonds [3,26], PLA absorbs higher water content (1.1%) than other polymers such as polypropylene (PP) or polyethylene (PE) [1,27]. The incorporation of untreated and treated sisal fibers into the PLA matrix clearly led to an increment in the water uptake values due to the higher hydrophilic character of lignocellulosic fibers. The water uptake at saturation values of composites increased with increasing the fiber content in the composite. Significant differences were not observed in water uptake profiles of composites based on fibers with different surface treatments. Zou *et al.* [8] studied the effect of different surface treatments on water absorption for composites based on PLA with 10 wt % of short sisal fiber. They treated sisal fibers with an alkali solution and with a combination of NaOH + silane chemical agent, where the used silane chemical agent was γ–amine propyltriehoxysilane. They observed that the water absorption of composites based on alkali treated fibers was similar to composites based on untreated fibers. However, in contrast to results obtained in this work, they also reported that the absorption of composites based on NaOH + silane fibers reduced about 22% compared to untreated fiber ones. A possible explanation not to observe this reduction in water uptake period of composites based on NaOH + silane treated fibers could be that the silane chemical agent and the treatment conditions used in this work were not the same. Zou *et al.* [8] soaked sisal fibers in 5% *w*/*v* γ–amine propyltriehoxysilane ethanol solution for 2 h, whereas, in this work, untreated and alkali treated sisal fiber were soaked in 3-(2-aminoethyl amino) propyltrimethoxy silane water solution for 3 h.

The experimentally determined parameters used in the calculations, including the initial linear slope of the sorption curve, θ, and equilibrium water uptake values, Δm(∞), are given in Table 1. The Fickian diffusion coefficient values of neat PLA matrix and composites with different fiber surface treatments and fiber content are also showed in Table 1. The equilibrium water uptake value of PLA matrix was small and the diffusion coefficient and corrected diffusion coefficient values of 6.7 × 10^−9^ cm^2^/s and 3.4 × 10^−9^ cm^2^/s were calculated, respectively. Obtained values are in agreement with those values published in the literature [28,29,30]. Yew *et al.* [28] immersed PLA samples in water at 30 °C for 30 days and they reported a diffusion coefficient value of 5.6 × 10^−9^ cm^2^/s. Deroine *et al.* [29] calculated a diffusion coefficient value of 48 × 10^−9^ cm^2^/s for PLA immersed in distilled water at 25 °C.

It was observed that increasing fiber loading in composite led to higher diffusion coefficient values. Due to the hydrophilic character of natural fibers, the inclusion of water molecules inside the composite materials was favored as demonstrated by the rate of the diffusion processes [31]. Yew *et al.* [28] prepared composites of PLA with 20 wt % rice starch and immersed them in water at 30 °C for 30 days. They observed that after adding rice starch to neat PLA, the diffusion coefficient value increased from 5.6 × 10^−6^ cm^2^/s to 6.9 × 10^−9^ cm^2^/s. Le Duigou *et al.* [30] prepared PLA composites with 20 wt % of flax fiber and immersed in seawater for two years. They observed that the corrected diffusion coefficient of PLA increased after the addition of flax fiber. Comparing different fiber surface treatments used, it was observed that the diffusion coefficient of composites based on NaOH + silane treated sisal fibers was reduced considerably, suggesting that this treatment seemed to be the most effective treatment to reduce water diffusion rate into the composites.

#### Effect of Water Uptake on Mechanical Properties

Table 2 shows the tensile strength (σ_t_), Young modulus (*E*_t_) and deformation at break (ε_b_) values of composites based on untreated and treated sisal fibers before and after water uptake period, respectively. After the immersion in distilled water at 28 °C for 209 days, tensile strength and Young modulus values decreased, probably because water molecules would change the structure and properties of the fibers, matrix and the interface between them. On the other hand, after the water absorption process, the values of deformation at break increased. Probably, water molecules acted as a plasticizer agent in composite material, leading to an increment of the maximum strain [32]. The decrease of tensile strength and Young modulus values after water uptake period was also reported by other authors for composites based on lignocellulosic fibers and polymeric matrices [1,33]. Islam *et al.* [33] studied the influence of hydrothermal ageing on mechanical properties of composites based on PLA with untreated and alkali treated hemp fibers. They observed a reduction in tensile strength and Young modulus values after samples were immersed in distilled water at 25 and 50 °C for three months. When fiber/matrix interface is accessible to moisture from the environment, the cellulosic fibers tend to swell, favoring the ultimate debonding of the fibers and resulting in a reduction in composites’ tensile strength values [16]. Moreover, hydrothermal ageing may also lead to the degradation of natural fibers and PLA matrix by hydrolytic mechanism. Consequently, water absorption and its resulting effects could contribute to the loss of the fiber/matrix adhesion, weakening the interface adhesion and also deteriorating the mechanical properties of fibers and PLA matrix [31].

The percentage variations of the tensile properties of studied systems after immersion in water are shown in Table 3. Taking into account the experimental results reported in Table 2 and Table 3, it was evident that the chemical treatments did not have noticeable effects on the retention of the mechanical properties.

**Figure 4:**
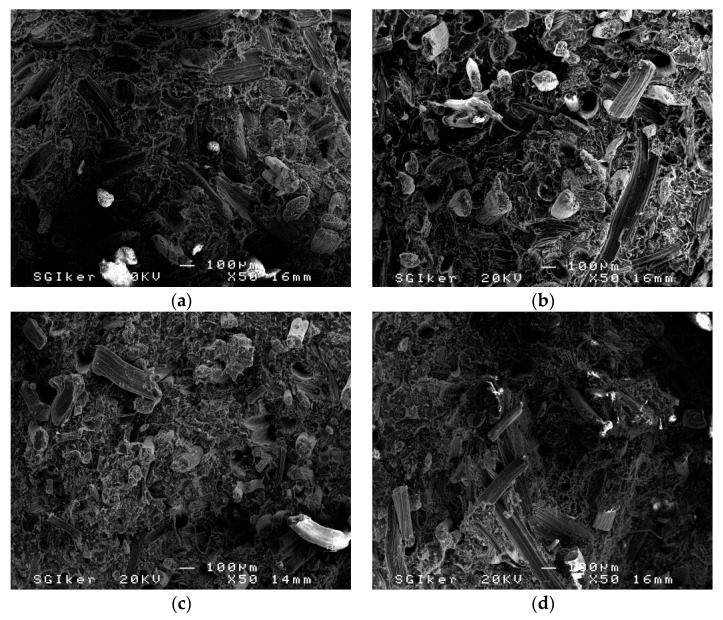
SEM micrographs of PLA/sisal composites (30 wt %) after water uptake period: (**a**) untreated; (**b**) silane; (**c**) NaOH; and (**d**) NaOH + silane.

In the previous work [5], SEM micrographs of composites before the water uptake period showed a homogeneous dispersion of sisal fibers in the PLA matrix. However, especially in composites based on untreated fibers and to a lesser extent in composites based on silane treated fibers, pulled-out fibers and holes were observed. On the other hand, pulled-out fibers could hardly be observed in SEM micrographs of alkali and NaOH + silane treated fibers, suggesting that sisal fibers were coated with polymer, and, consequently, fiber/PLA adhesion were improved. SEM micrographs of composites after the water uptake period are showed in Figure 4a–d. The micrographs showed that the dispersion of sisal fibers was homogenous in the PLA matrix. Moreover, it was observed that the sisal fibers were randomly oriented in the matrix. However, in all systems, pulled-out fibers and holes can be observed, suggesting that water seemed to damage the adhesion between the PLA matrix and sisal fibers. Fiber/PLA poor adhesion caused either an inefficient stress transfer from matrix to fiber, worsening composites’ mechanical properties, and also an easy penetration and storage of water. Therefore, all studied composites showed similar water absorption profiles.

### 3.3. Young Modulus Prediction

Table 4 shows the mean length, diameter and aspect ratio values of sisal fibers after processing. Moreover, *E*_m_ and *E*_f_ values of untreated and treated sisal fibers obtained in the previous works are given in Table 4 [3,5]. All of these values were used to predict the Young modulus values of PLA/sisal fiber composites.

**Figure 5:**
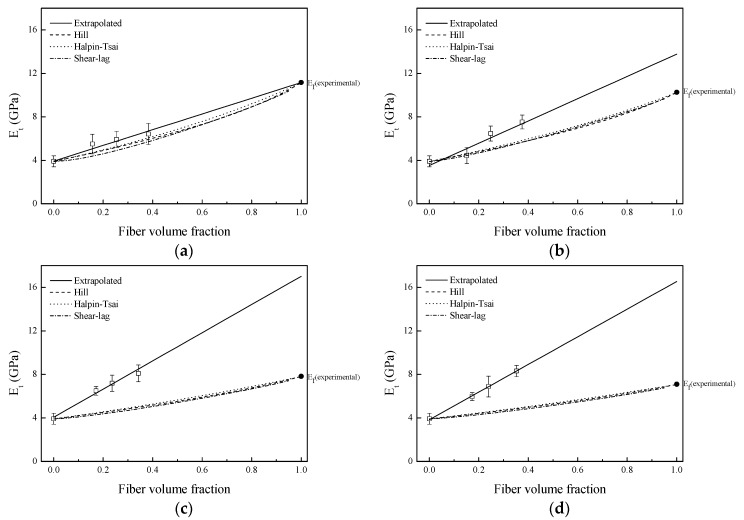
Estimation of composite modulus values using fiber experimental Young modulus values: (**a**) untreated; (**b**) silane; (**c**) NaOH; and (**d**) NaOH + silane.

Figure 5a–d compares the theoretical and experimental tensile modulus values of composites based on untreated and treated sisal fibers. It was observed that the experimental tensile modulus of composites increased linearly as the content of fiber was increased [5].

**Figure 6:**
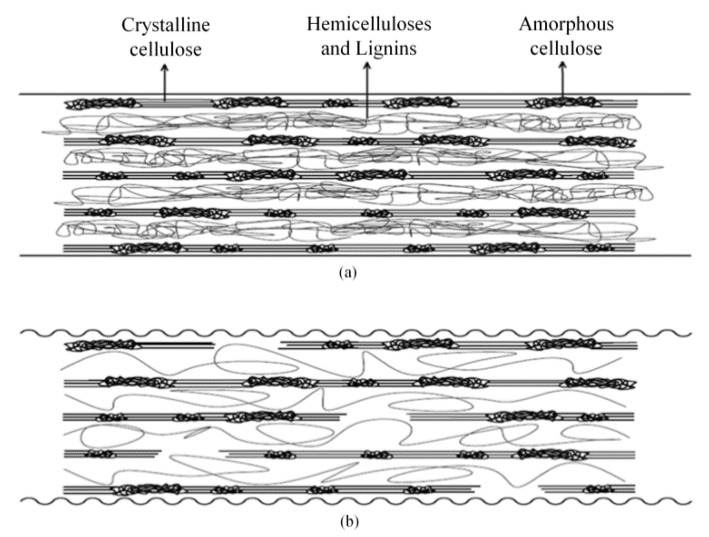
Schematic illustration showing the hypothetical effect of alkali treatment on the sisal fiber structure: (**a**) untreated fiber; and (**b**) alkali treated fiber.

For composites based on untreated sisal fibers and PLA matrix, the correlation between theoretical and experimental values was good, whereas for composites based on silane treated fibers, the correlation between theoretical and experimental values was not very good. The experimental Young modulus values of composites based on alkali and NaOH + silane treated fibers were considerably much higher than theoretical ones, and it was decided to fit the experimental Young modulus values of composites to a straight line. At fiber volume fraction of one, which means that there would not be a matrix, the extrapolated fiber modulus values, *E*_f_ (extrapolated), were obtained and reported in Table 4. In composites based on untreated sisal fibers, no difference between the extrapolated and experimental modulus values was observed. However, in composites based on treated sisal fibers, especially in alkali and NaOH + silane treated fibers, significant differences can be observed. Although alkali treatment removed non-cellulosic constituents and increased the cellulose fraction of sisal fibers [5], it was observed that the obtained tensile properties of alkali and NaOH + silane treated fibers were lower than untreated ones, suggesting that alkali treatment could create voids in the fiber structure [3,5]. The schematic drawing of the hypothetical effect of alkali treatment on fiber structure is shown in Figure 6.

**Figure 7:**
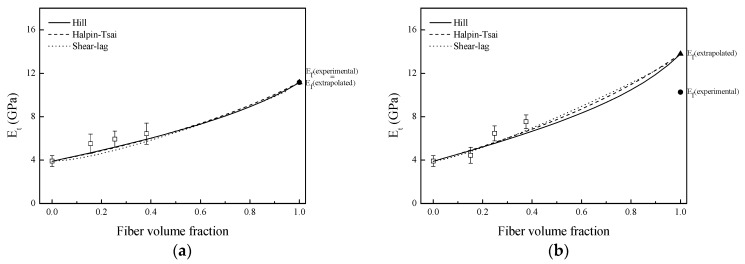

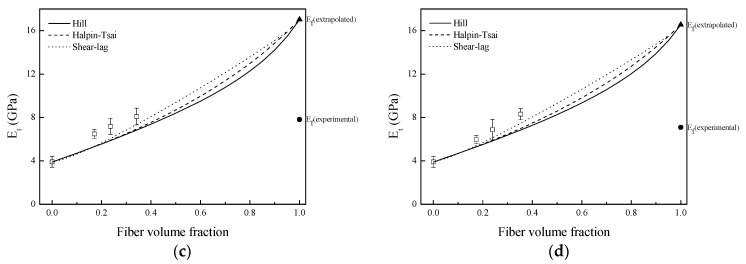
Determination of composite modulus values with extrapolated Young modulus values of untreated and treated sisal fibers: (**a**) untreated; (**b**) silane; (**c**) NaOH; and (**d**) NaOH + silane.

It seemed that the created voids were responsible for obtaining the lowest Young modulus values determined by tensile test. However, previous work suggested that these voids could be filled during the injection process with melted PLA, improving fiber/PLA mechanical interlocking adhesion [5]. On the other hand, as the cellulose fraction in alkali and NaOH + silane treated fibers was higher than in untreated fibers ones, composites based on these fibers showed the highest values of tensile modulus, as observed in Figure 7a–d. In summary, experimental fiber tensile modulus values used for composites prediction were suitable for untreated fibers and to a lesser extent for silane treated fibers. However, experimental tensile moduli used for alkali and NaOH + silane fibers were not suitable for predicting the composites modulus values because the experimental composite modulus values were significantly higher than estimated ones. Alkali treatment removed hemicelluloses and lignins from sisal fiber, leading to creation of voids in fiber structure. Therefore, the moduli values estimated for composites were for composites with fibers that had internal voids. Nevertheless, during the injection molding process, because high injection pressure was applied, melted PLA can flow inside these voids. As a result, obtained experimental composite modulus values were significantly higher than estimated ones. Therefore, the *E*_f_ (extrapolated) values of alkali and NaOH + silane treated fibers are more accurate for composite modulus prediction than values determined by tensile test (Figure 7c,d).

## 4. Conclusions

All fiber surface treatments delayed the water absorption for a short period of time. For this reason, there was not a big difference in composites’ water uptake profiles after different surface treatments. However, the diffusion coefficients of composites based on NaOH + silane treated sisal fibers were reduced considerably, suggesting that this treatment seemed to be the most effective treatment to reduce the water diffusion rate into the composites. Regarding the mechanical properties, tensile strength and Young modulus values of composites after the water uptake period were decreased due to the loss of sisal fiber/PLA adhesion as observed in SEM images. Experimental fiber tensile modulus values used for composite prediction were suitable for untreated, and, to a lesser extent, for silane treated fibers. However, experimental tensile moduli used for alkali and NaOH + silane fibers were not suitable for predicting the composite moduli. Nevertheless, *E*_f_ (extrapolated) values of alkali and NaOH + silane treated fibers are more accurate for composite modulus prediction than values determined by tensile test.

## Figures and Tables

**Table 1:** Fiber volume fraction (${Ø}_{f}$), initial linear slope (θ), equilibrium water uptake (Δm(∞)), diffusion coefficient (*D*) and corrected diffusion coefficient (*D*_c_) values for poly(lactic acid) matrix and composites.

Sample	${Ø}_{f}$	θ (%/min^1/2^)	Δm(∞)(%)	*D* × 10^−9^ (cm^2^/s)	*D*_c_ × 10^−9^ (cm^2^/s)
PLA	0	0.00479	1.10	6.7	3.4
Untreated	0.156	0.02869	5.26	10.5	5.4
	0.254	0.04547	7.83	11.9	6.1
	0.382	0.06518	10.08	14.8	7.6
Silane	0.152	0.03150	5.41	12.0	6.2
	0.248	0.04595	8.12	11.3	5.8
	0.375	0.06135	10.31	12.5	6.4
NaOH	0.171	0.03143	5.33	12.3	6.3
	0.236	0.03761	6.26	12.4	6.4
	0.341	0.05057	8.87	12.7	6.5
NaOH + silane	0.174	0.02364	4.97	8.0	4.1
	0.239	0.03768	7.41	9.2	4.7
	0.345	0.05352	9.51	11.2	5.7

**Table 2:** Tensile properties of poly(lactic acid) matrix and composites based on sisal fibers before and after water uptake period.

Sample	Before Water Uptake Period				After Water Uptake Period		
	${Ø}_{f}$	σ_t_ (MPa)	*E*_t_ (GPa)	ε_b_ (%)	σ_t_ (MPa)	*E*_t_ (GPa)	ε_b_ (%)
PLA	0	66.1 ± 1.7	3.9 ± 0.5	2.4 ± 0.3	62.9 ± 1.5	3.5 ± 0.3	2.8 ± 0.1
Untreated	0.156	53.8 ± 0.9	5.5 ± 0.9	1.8 ± 0.4	48.1 ± 1.6	3.2 ± 0.4	3.1 ± 0.1
	0.254	47.3 ± 1.7	5.9 ± 0.7	1.1 ± 0.3	40.4 ± 1.8	4.0 ± 0.1	2.2 ± 0.1
	0.382	39.3 ± 3.4	6.4 ± 1.0	0.8 ± 0.1	35.6 ± 1.4	1.5 ± 0.2	2.2 ± 0.1
Silane	0.152	57.6 ± 2.9	4.4 ± 0.7	1.9 ± 0.3	33.8 ± 2.2	2.8 ± 0.3	2.5 ± 0.1
	0.248	59.9 ± 1.5	6.4 ± 0.7	1,5 ± 0.3	34.5 ± 2.0	2.7 ± 0.1	1.9 ± 0.1
	0.375	61.8 ± 3.1	7.5 ± 0.6	1.0 ± 0.2	32.0 ± 1.9	1.6 ± 0.2	1.6 ± 0.2
NaOH	0.171	66.1 ± 3.2	6.5 ± 0.4	1.6 ± 0.3	45.4 ± 1.0	3.3 ± 0.2	2.4 ± 0.3
	0.236	74.8 ± 3.5	7.2 ± 0.7	1.5 ± 0.1	35.1 ± 0.4	3.0 ± 0.2	2.3 ± 0.3
	0.341	81.5 ± 1.4	8.1 ± 0.8	1.4 ± 0.1	31.9 ± 1.3	2.1 ± 0.5	2.4 ± 0.1
NaOH + silane	0.174	66.9 ± 1.0	5.9 ± 0.4	1.8 ± 0.2	42.3 ± 0.9	3.5 ± 0.1	3.2 ± 0.3
	0.239	74.7 ± 1.4	6.9 ± 0.9	1.5 ± 0.1	38.0 ± 1.3	2.9 ± 0.2	2.0 ± 0.2
	0.345	78.6 ± 3.5	8.3 ± 0.5	1.4 ± 0.2	34.9 ± 2.0	2.7 ± 0.3	1.9 ± 0.2

**Table 3:** Percentage variations of the tensile properties of studied systems after immersion in water.

Sample	${Ø}_{f}$	Δσ_t_ (MPa)	Δ *E*_t_ (GPa)	Δε_b_ (%)
PLA	0	−5	−10	+17
Untreated	0.156	−11	−42	+72
	0.254	−15	−32	+100
	0.382	−9	−77	+175
Silane	0.152	−41	−36	+32
	0.248	−42	−58	+27
	0.375	−48	−79	+60
NaOH	0.171	−31	−49	+50
	0.236	−53	−58	+53
	0.341	−61	−74	+71
NaOH + silane	0.174	−37	−41	+78
	0.239	−49	−58	+33
	0.345	−59	−67	+36

**Table 4:** Mean length, diameter, aspect ratio (*l*/*d*), extrapolated and experimental Young modulus values of untreated and treated sisal fibers used for predicting composites Young modulus.

Fiber	Length (μm)	Diameter (μm)	*l*/*d*	*E*_f_ (Experimental) (GPa)	*E*_f_ (Extrapolated) (GPa)
Untreated	513	130	3.9	11.1	11.2
Silane	571	104	5.5	10.3	13.8
NaOH	543	94	5.8	7.8	17.0
NaOH + silane	555	84	6.6	7.1	16.5
